# Continuous warming shift greening towards browning in the Southeast and Northwest High Mountain Asia

**DOI:** 10.1038/s41598-021-97240-4

**Published:** 2021-09-09

**Authors:** Yongchang Liu, Zhi Li, Yaning Chen

**Affiliations:** 1 State Key Laboratory of Desert and Oasis Ecology, Xinjiang Institute of Ecology and Geography, Chinese Academy of Sciences, Urumqi, 830011 China; 2 University of the Chinese Academy of Sciences, Beijing, 100049 China

**Keywords:** Climate-change ecology, Climate-change ecology

## Abstract

Remote sensing and ground vegetation observation data show that climate warming promotes global vegetation greening, and the increase in air temperature in High Mountain Asia (HMA) is more than twice the global average. Under such a drastic warming in climate, how have the vegetation dynamics in HMA changed? In this study, we use the Normalized Difference Vegetation Index (NDVI) from 1982 to 2015 to evaluate the latest changes in vegetation dynamics in HMA and their climate-driving mechanisms. The results show that over the past 30 years, HMA has generally followed a “warm-wet” trend, with temperatures charting a continuous rise. During 1982–1998 precipitation increased (1.16 mm yr^−1^), but depicted to reverse since 1998 (− 2.73 mm yr^−1^). Meanwhile, the NDVI in HMA increased (0.012 per decade) prior to 1998, after which the trend reversed and declined (− 0.005 per decade). The main reason for the browning of HMA vegetation is the dual effects of warming and precipitation changes. As mentioned, the increase in air temperature in HMA exceeds the global average. The increase of water vapor pressure deficit caused by global warming accelerates the loss and consumption of surface water, and also aggravates the soil water deficit. That is to say, the abnormal increase of land evapotranspiration far exceeds the precipitation, and the regional water shortage increases. Climate change is the primary factor driving these vegetation and water dynamics, with the largest proportion reaching 41.9%.

## Introduction

It is widely expected that a warmer climate promotes vegetation activities^1–4^. Generally, these responses can be grasped from the vegetation index. For example, multiple long-term satellite Leaf Area Index (LAI) data sets and Normalized Difference Vegetation Index (NDVI) data sets indicate that the vegetation greening trend in the high latitudes of the northern hemisphere has increased significantly, and that the vegetation greening period is occurring earlier in most parts of High Mountain Asia (HMA)^5–8^.

However, for some regions, the higher temperatures are causing water deficits in the absence of available precipitation and surface water. Due to significant warming and other factors, global evapotranspiration has increased at a rate of 0.88 mm yr^−2^ over the past 30 years, and the growth rate in the northern hemisphere is about 1.5 times that of the southern hemisphere^9,10^. The continued warming has had a negative impact on vegetation. Satellite data show that vegetation browning has appeared in partial parts of the world since 2000^11,12^, with several studies reporting that excessive vegetation transpiration, soil water evaporation, and excessive water vapor pressure are jointly affecting vegetation water deficit and carbon dioxide fertilization efficiency, restricting vegetation growth^13–17^.

**Figure 1:**
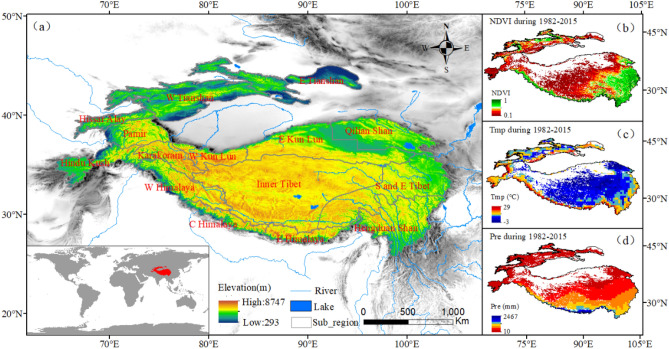
Study area. (**a**) High Mountain Asia (HMA), which mainly includes the Tibetan Plateau (TP), Tianshans, Pamir Plateau, Kunlun Mountains and Qilian Mountains. Spatial distribution of (**b**) NDVI, (**c**) air temperature (Tmp) and (**d**) precipitation (Pre) in HMA growing season from 1982 to 2015. Data sources: NASA and CRU (Generated by ArcGIS 10.3, URL: http://www.esri.com/software/arcgis/arcgis-for-desktop).

HMA, with an average altitude of 4000 m, covers approximately 4 × 10^6^ km^2^. It is located between the South Asian subcontinent and Central Asia, with a geographical range of 25°N–51°N to 64°E–106°E (Fig. 1)^18^. It consists of a large number of the highest mountains and plateaus in the world (such as Tianshan, Himalayas, Hindu Kush, Karakoram, Pamir, Qilian, and Hengduan Mountains). In addition, HMA is considered to be the "Third Pole" or "Asian Water Tower" in Asia and even the world^19^, which is the birthplace of the main rivers in East and South Asia providing water for them. These rivers supply water to more than 1 billion people living in or near the region, which is an important ecological security barrier^19,20^. This area has typical Alpine arid climate and vegetation types, mainly including alpine grassland, alpine meadow, temperate grassland, deciduous broad-leaved forest and forest. The alpine grassland is about 152.15 × 10^4^ km^2^. It is one of the important pastoral areas in Asia, especially in China. At the same time, the ecological environment in HMA is fragile and sensitive to climate change^21–23^. Generally speaking, Indian and Asian monsoon in summer and westerly belt in winter are the main sources of water vapor in HMA^24^. Since the 1970s, HMA has experienced drastic environmental changes that includes a rise in air temperature rate of more than twice the global average^25^. The continuous warming has caused significant changes in precipitation patterns, with the precipitation rate is about 1.43 mm yr^−1^ ^24^.

The overall trend of “warm-wet” has promoted the greening of HMA vegetation, and recent research has found that warming has a positive effect on vegetation growth in spring and winter^26,27^. Simulated warming experiments also show that warming has a positive impact on alpine vegetation growth^28^. Based on these findings, we can assume that a suitable temperature rise is indeed beneficial for promoting vegetation growth^29^. However, the data indicate that as the temperature continues to rise, the beneficial factors that climate warming brings to plant growth in high latitude regions may be weakening and even starting to inhibit vegetation growth^10^.

Alpine vegetation is generally tolerant to low temperatures but sensitive to high ones^23^. Piao et al.^30^ found that warming, when it occurs in the fall, inhibits vegetation growth. According to station data, HMA’s highest monthly temperature can reach 15 °C^24^, which exceeds the optimum temperature (10 °C) for vegetation growth in high latitude and mountainous areas^31^. In addition, recent studies have discovered that with the intensification of global warming, the impact of air temperature on vegetation growth is weakening, leaving precipitation as the main influencing factor of vegetation dynamics^30,32,33^. In arid regions, an increase in precipitation can reduce the negative effects of evapotranspiration to a certain extent, while a decrease in precipitation can aggravate the water stress caused by warming^34–36^. Although the current conclusions on the correlations between vegetation growth and climatic factors are inconsistent, it is clear that temperature and precipitation—the two key components of climate change—have a dual and superimposed effect on vegetation growth. The increasing influence of these climatic factors is resulting in the development of a complex and uncertain relationship between climate change and vegetation.

The global warming trend is expected to continue into the future. Using the current climate trajectory as a guide, the temperatures across most of HMA will rise by 2.8–4.9 °C by the end of this century and precipitation will increase by 15–21% during the same timeframe^37^. However, due to the fluctuations of atmospheric circulation and monsoons as well as the influence of altitude, the temporal and spatial distribution of precipitation in HMA will vary greatly. There may also be a certain tendency towards localized drought in some areas^24,38,39^. Based on historical, current and forecast climatic conditions, climate warming and its material and energy cycle effects have brought new challenges to the stability of regional ecosystems^27,40^. In this context, the response mechanism of ecosystems in HMA to climate change has become an important feature of current global climate change research^41^. Identifying which climatic factors are most sensitive to changes in the plateau vegetation at the regional scale, and which climatic factors can most effectively influence the growth and distribution of vegetation, will vary from place to place.

As a complete and unique macrophysical-geographic ecosystem^42,43^, HMA serves not only as the main driving force in global climate change, but also as its representative response that may be generalized and applied to other regions around the world^44,45^. However, as of now, research on the response mechanism of vegetation dynamics to climate change is limited and needs to be deepened^22^, as understanding these change mechanisms is of great significance for quantitatively predicting future vegetation-climate responses and feedback mechanisms^46^.

## Results

### Changes in NDVI of HMA

#### Time change

**Figure 2:**
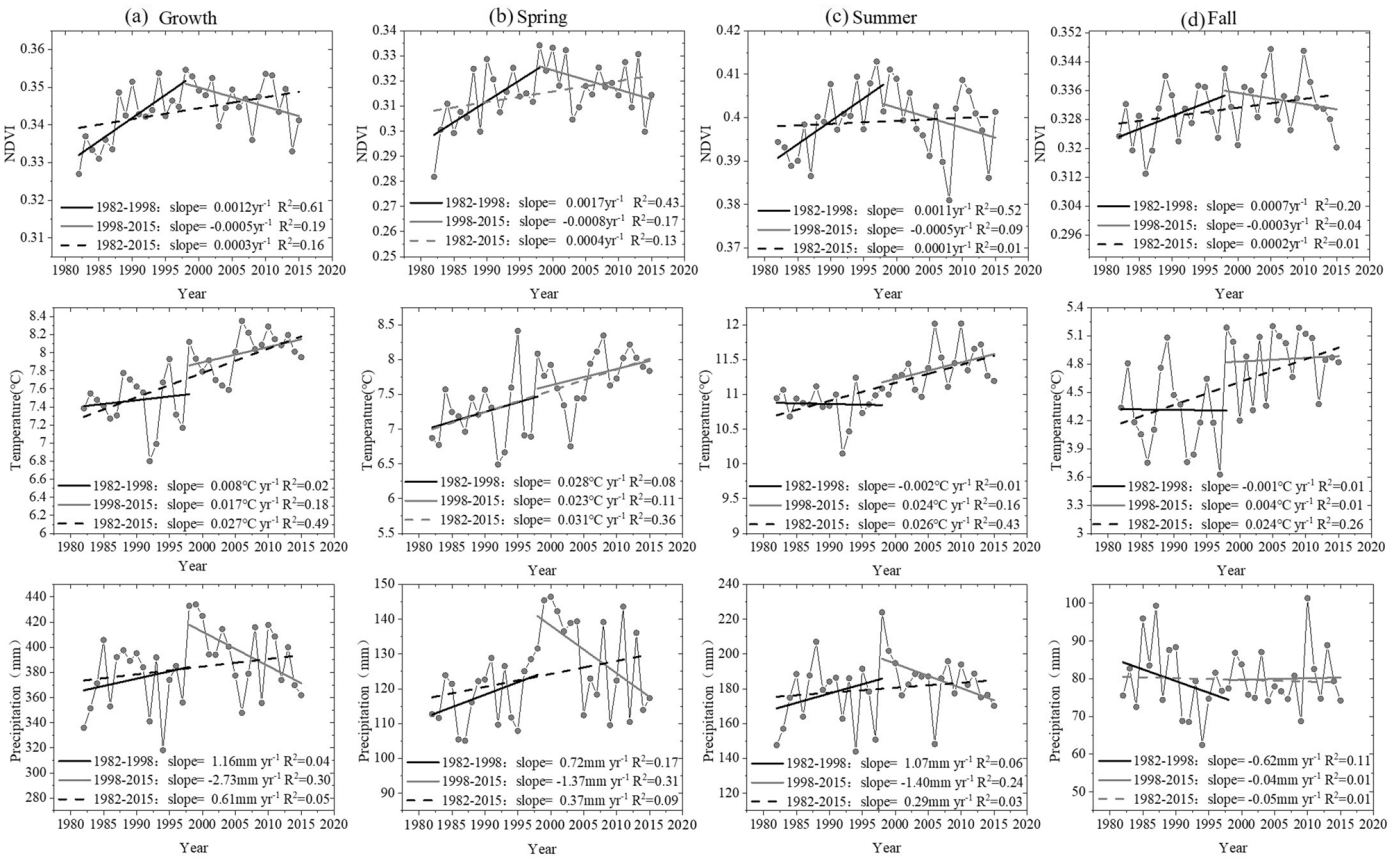
Inter-annual dynamics of growing season NDVI, air temperature, and precipitation in HMA from 1982 to 2015 (Generated by Origin 2020b, URL: https://www.originlab.com/).

Over the past 34 years, the growing season NDVI (NDVI_GS_) of vegetation in HMA has generally shown an upward trend at a rate of 0.003 per decade (Fig. 2). However, there was a reversal change around 1998, which was mainly manifested as a significant upward trend from 1982 to 1998 (rising rate of 0.012 per decade) and a downward trend after 1998 (falling rate of − 0.005 per decade). These trends indicate that the NDVI of HMA first increased and then decreased, and that the vegetation showed a browning trend against a background of overall greening.

On a seasonal scale, the NDVI also showed similar changes around 1998. Specifically, in the first period (1982–1998), the growth rate of NDVI ( 0.017 per decade) was the largest in spring (May–June, SP) and the smallest (0.007 per decade) in fall (September–October, FA). In the second period (1998–2015), the NDVI decrease rate was the largest in spring (− 0.008 per decade), and the smallest in fall (− 0.003 per decade). For the whole period under study, the growth rate of NDVI was the largest in spring (0.004 per decade), while the growth rate of NDVI was the smallest (0.001 per decade)in summer (July–August, SU). In the dynamic changes and trend reversal of NDVI, spring and summer exhibited a greater contribution rate, which is in line with the current research conclusions on phenology and vegetation greening trends^35,47^.

#### Spatial changes

**Figure 3:**
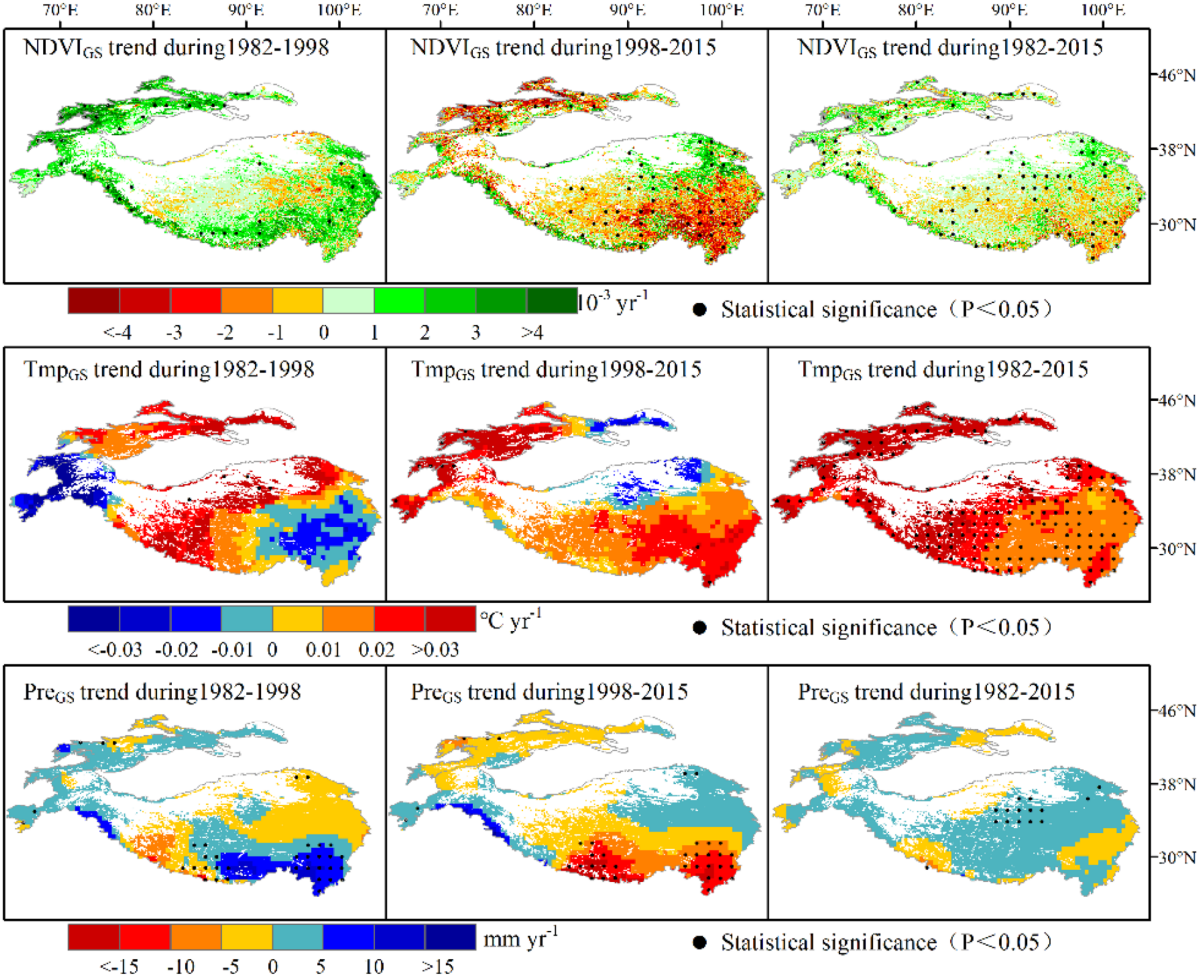
Spatial distribution of growing season (May–October) NDVI trends for air temperature and precipitation in HMA from 1982 to 2015. Note that regions with black dots indicate that trend values are statistically significant (p < 0.05) (Generated by ArcGIS 10.3, URL: http://www.esri.com/software/arcgis/arcgis-for-desktop).

From 1982 to 2015, the NDVI_GS_ in 67.4% of HMA vegetation pixels showed an increasing trend. As can be seen in Fig. 3, the average change rate was 3.34 × 10^−4^ yr^−1^ and vegetation browning were mainly distributed in the southeast. Of all the NDVI_GS_ examined here, those from 1982 to 1998 display a more significant greening trend, with an average change rate of 1.2 × 10^−3^ yr^−1^ and a greening area accounting for 84.4%. The vegetation browning during this time period is scattered throughout the middle and eastern portions of the TP. After 1998, the vegetation starts into a browning trend, with an average rate of change of − 4.4 × 10^−4^ yr^−1^ and the browning area accounting for 59.4%. During this time period, the greening phenomenon was mainly distributed in the northeast.

Research indicates that vegetation growth is strongly seasonal^48^. The seasonal variability has the characteristics of spring > fall > summer numerically, which is similar to the conclusion of Wang^46^. From the perspective of geographical distribution, the areas where vegetation NDVI increased in spring prior to 1998 were Tianshans, the surrounding Himalayas, and Hengduans. Areas where NDVI increased in summer were mainly central and western HMA. In fall, prior to 1998, NDVI increased mainly in northwest HMA and the eastern and southern TP. After 1998, most of the areas showed a browning trend. However, some areas did exhibit a greening trend, such as Hindu Kush in spring, northeast HMA in summer, and the Himalayan circum region, eastern Kunlun Mountains, and Qilian Mountains in fall.

### Dynamic driving analysis of HMA vegetation

#### Temporal and spatial changes of temperature and precipitation

From 1982 to 2015, the overall performance of the HMA growing season was a "warm-wet" trend. The temperature change rate was 0.027 °C yr^−1^ and the precipitation change rate was 0.61 mm yr^−1^, with temperature and precipitation showing different change patterns. Specifically, after 1998, temperature showed a sudden increase and the rate changed from 0.008 to 0.017 °C yr^−1^. However, from 1998 onward, precipitation showed a reverse change that first rose and then fell, with the rate changing from 1.16 to − 2.73 mm yr^−1^. Even in fall, precipitation exhibited a continuous decreasing trend (Fig. 2).

The changing patterns of temperature and precipitation in the other seasons are similar to those in the growing season. From 1982 to 2015, the changing rates of temperature and precipitation in each season were 0.031 °C yr^−1^, 0.026 °C yr^−1^, 0.024 °C yr^−1^, 0.37 mm yr^−1^, 0.29 mm yr^−1^, and − 0.05 mm yr^−1^. Further analysis found that the temperature and precipitation of each season also turned in 1998, and there are significant regional differences in the reversals of temperature and precipitation trends (Fig. 3, Figure S1-3). Taking 1998 as the boundary, during the growing season, southeast and northwest HMA experienced a warming process with decreasing precipitation, while northeast HMA showed a cooling process with increasing precipitation. In the spring, the western and southern regions underwent a warming process with decreasing precipitation, and the northeast experienced a cooling process with increasing precipitation. In summer, the northeast showed a cooling process with decreasing precipitation, the southwest had a warming process with increasing precipitation. In fall, areas west of 90°E in HMA underwent a cooling process with increased precipitation, whereas areas east of 90°E experienced a warming process with increased precipitation.

#### Correlation analysis of NDVI, temperature, and precipitation

**Figure 4:**
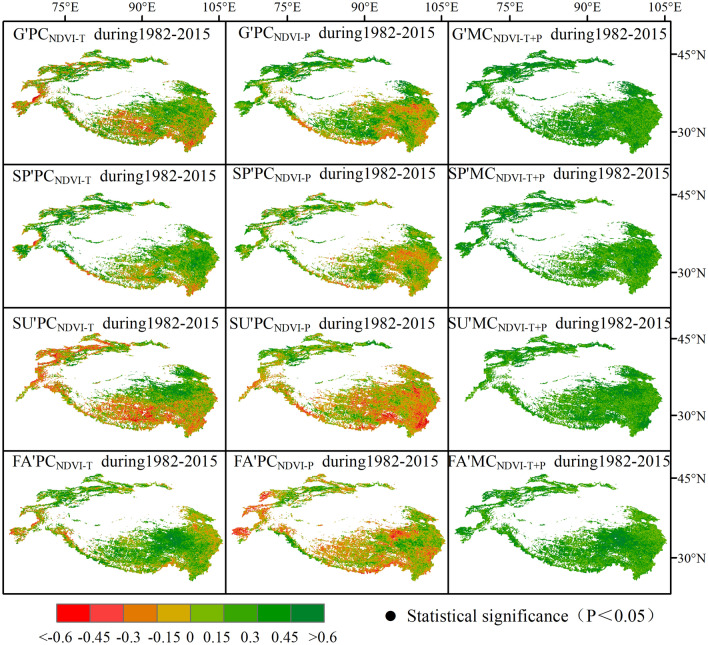
Spatial distribution of inter-annual partial correlation coefficients between NDVI and precipitation (PC_NDVI-P_) and temperature (PC_NDVI-T_) and multiple correlations between NDVI, temperature and precipitation (MC_NDVI−T+P_) for growing seasons (G), i.e. spring (SP), summer (SU) and fall (FA), during 2000–2018 in HMA. Note that regions with black dots indicate that partial correlation and multiple correlation coefficients are statistically significant (p < 0.05) (Generated by ArcGIS 10.3, URL: http://www.esri.com/software/arcgis/arcgis-for-desktop).

Autocorrelation function (ACF) analysis was performed to check whether there is autocorrelation in NDVI, since this may affect the correlation between NDVI with temperature and precipitation. Findings revealed that the ACF values of NDVI series fluctuate within the positive and negative double standard error. This implies that NDVI can be understood as a random sequence, so it can be used for subsequent correlation analysis. Therefore, correlation analysis indicated that the multiple correlations between NDVI with temperature and precipitation at each time scale are relatively large, and that vegetation dynamics are significantly affected by temperature and precipitation (Fig. 4). The partial correlation coefficients between NDVI and temperature are mostly positive. NDVI and temperature are negatively correlated in parts of the growing season, which are distributed in western and southern HMA. In summer, NDVI showed a significant negative correlation with temperature, which was allocated to northwestern and southern HMA. The temperature increases that occurred in spring and fall promoted the extension of the growing season and the accumulation of biomass by affecting the phenological process of vegetation, while the temperature increases in summer restricted the growth of vegetation through the physiological process of plants and the atmospheric environment.

Our study shows that the NDVI is mostly negatively correlated with precipitation. However, across HMA, spring, summer and fall were positively correlated in the northwest, while summer and fall were mostly negatively correlated in the southeast. Abundant precipitation can offset the negative effects of warming in spring and summer and promote vegetation growth together with temperature. However, if the precipitation base changes from humid to relatively dry, the drought stress caused by warming is aggravated and becomes the main factor restricting vegetation growth.

In addition, the change trend of temperature and precipitation showed many similarities with NDVI. Firstly, the temperature and precipitation in the growing season from 1982 to 2015 in HMA charted an overall upward trend (0.027 °C yr^−1^, 0.61 mm yr^−1^) (Fig. 2a). Secondly, both of them experienced a trend reversal in 1998, when the temperature change trend leapt and the precipitation change trend reversed. Prior to the jump in temperature, warming and wetting corresponded to vegetation greening in northwest, south-central and eastern HMA. However, after the jump in temperature, warming and drying corresponded to vegetation browning in northwest, central and southern HMA, while cooling and wetting corresponded to vegetation greening in the Qilian Mountains.

On a seasonal scale, after 1998, the amount of browning in spring was low in central HMA and high in the northwest and southeast HMA, which is the same as the warming and drought trend. Prior to 1998, summer precipitation in the eastern part of HMA showed an increasing tendency. At the same time, the spatial distribution of temperature and NDVI variability was comparable. After 1998, the temperature in the northeastern part of HMA charted a downward trend, while precipitation increased and NDVI increased. Temperature in the southeast and Tianshans area increased, precipitation decreased, and NDVI decreased.

Prior to 1998, in fall, the warming and drying in the Tianshans corresponded to vegetation greening, the cooling and drying of the TP corresponded to vegetation browning. After 1998, however, the warming and wetting of the Tianshans corresponded to the browning of vegetation, the warming and wetting of the TP corresponded to the greening of vegetation. Therefore, although temperature in spring and fall had a greater effect on plant growth, a jump in temperature made precipitation the key factor restricting vegetation growth. This shows that the dynamic changes of vegetation under global climate change are not simply dominated by temperature or precipitation, but are influenced by both of these factors at different time scales.

#### Coupling relationship between NDVI and water deficit

**Figure 5:**
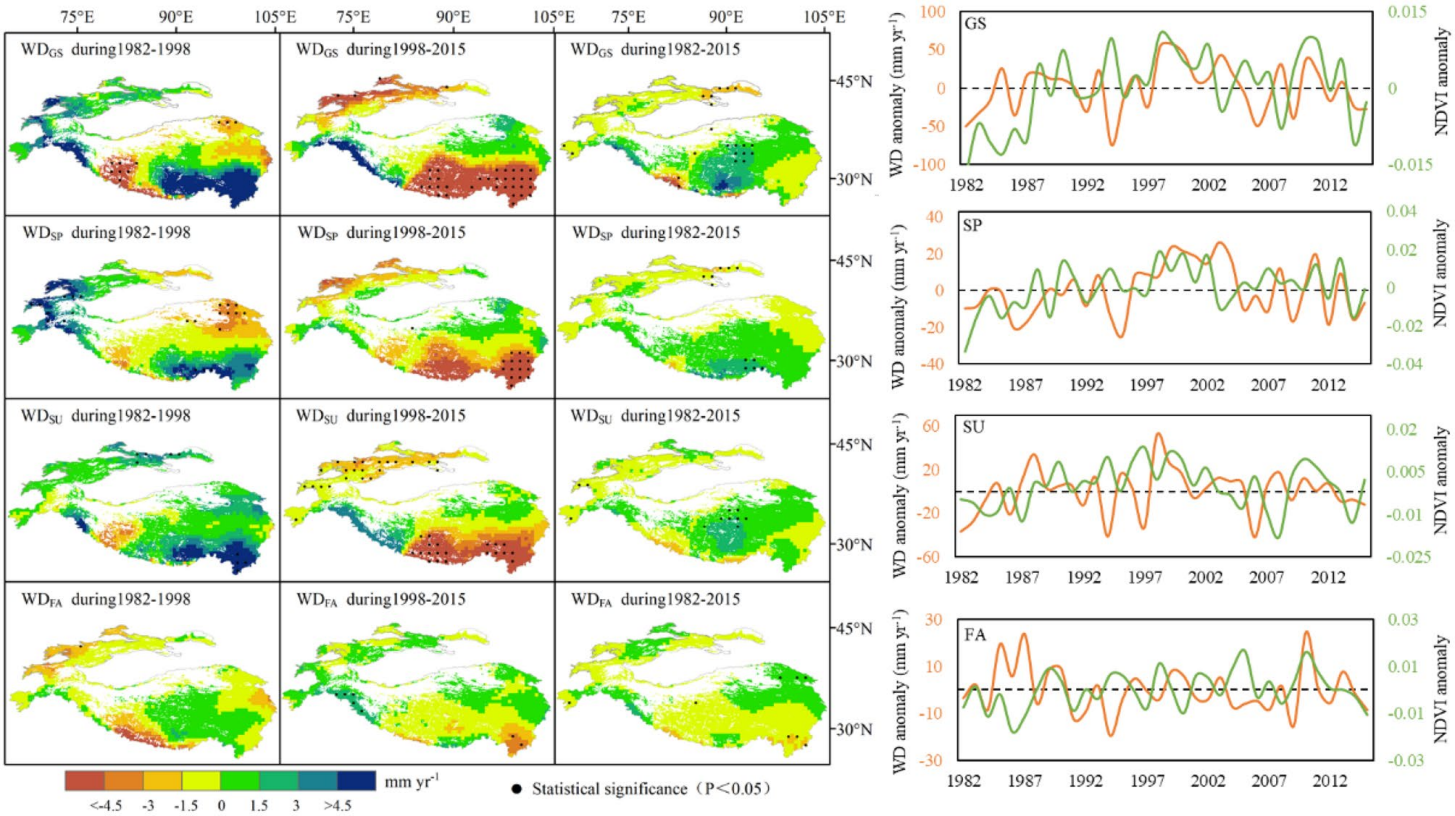
Spatial distribution of trends and inter-annual dynamics of water deficit for growing seasons (WD_GS_), spring (WD_SP_), summer (WD_SU_) and fall (WD_FA_) during 1982–2015 in HMA. Note that regions demarcated by black dots indicate trend values that are statistically significant (p < 0.05) (Generated by ArcGIS 10.3, URL: http://www.esri.com/software/arcgis/arcgis-for-desktop, and Matlab R2013a, URL: http://cn.mathworks.com/products/matlab/).

The water deficit can comprehensively represent the combination of regional temperature and water (Fig. 5). During our study period, the water deficit shows an increasing trend, accounting for 52.9% of HMA, with an average rate of 0.127 mm yr^−1^. This rate is consistent with the proportion and distribution of greening pixels (67.4%), even though the precipitation variability was about 5 times the rate of water deficit. Prior to 1998, the water deficit in HMA (61.9%) showed an increasing trend, with an average rate of 1.341 mm yr^−1^. However, after 1998, 70.1% of HMA experienced a decreasing water deficit trend at an average rate of − 2.653 mm yr^−1^.

On a long-term scale, the continuous warming in the past 30 years has aggravated the regional water cycle. Taking 1998 as the boundary, the land evapotranspiration caused by warming has far exceeded precipitation, reversing the trend of regional water deficit changes. For the period under study, 57.1% and 57.3% of the pixels exhibited an increasing trend of water deficit during spring and summer, with an average rate of change of 0.144 mm yr^−1^ and 0.096 mm yr^−1^, respectively. The corresponding green pixels were 65.6% and 56.8%, respectively. Before 1998, 52.2% and 85.2% of HMA showed an increasing water deficit trend, with an average change rate of 1.065 mm yr^−1^ and 1.075 mm yr^−1^ in spring and summer, respectively. After 1998, 72.8% and 75.0% of the study area charted a decreasing trend, with an average change rate of − 1.357 mm yr^−1^ and − 1.453 mm yr^−1^ for spring and summer, respectively. A reversal of this trend occurred in northwest and south-central HMA, similar to the distribution of vegetation dynamics.

However, the changing water deficit trend for fall showed different patterns. Between 1982 and 1998, the water deficit for 62.3% of HMA indicated a decreasing trend, with an average rate of − 0.799 mm yr^−1^. After that, the water deficit showed a clear upward tendency. Specifically, the proportion dropped to 53.2% and the average rate turned positive to 0.157 mm yr^−1^. The areas where the trend reversal occurred were mainly located in the northwest and eastern HMA.

#### Quantitative analysis of vegetation driving force

**Figure 6:**
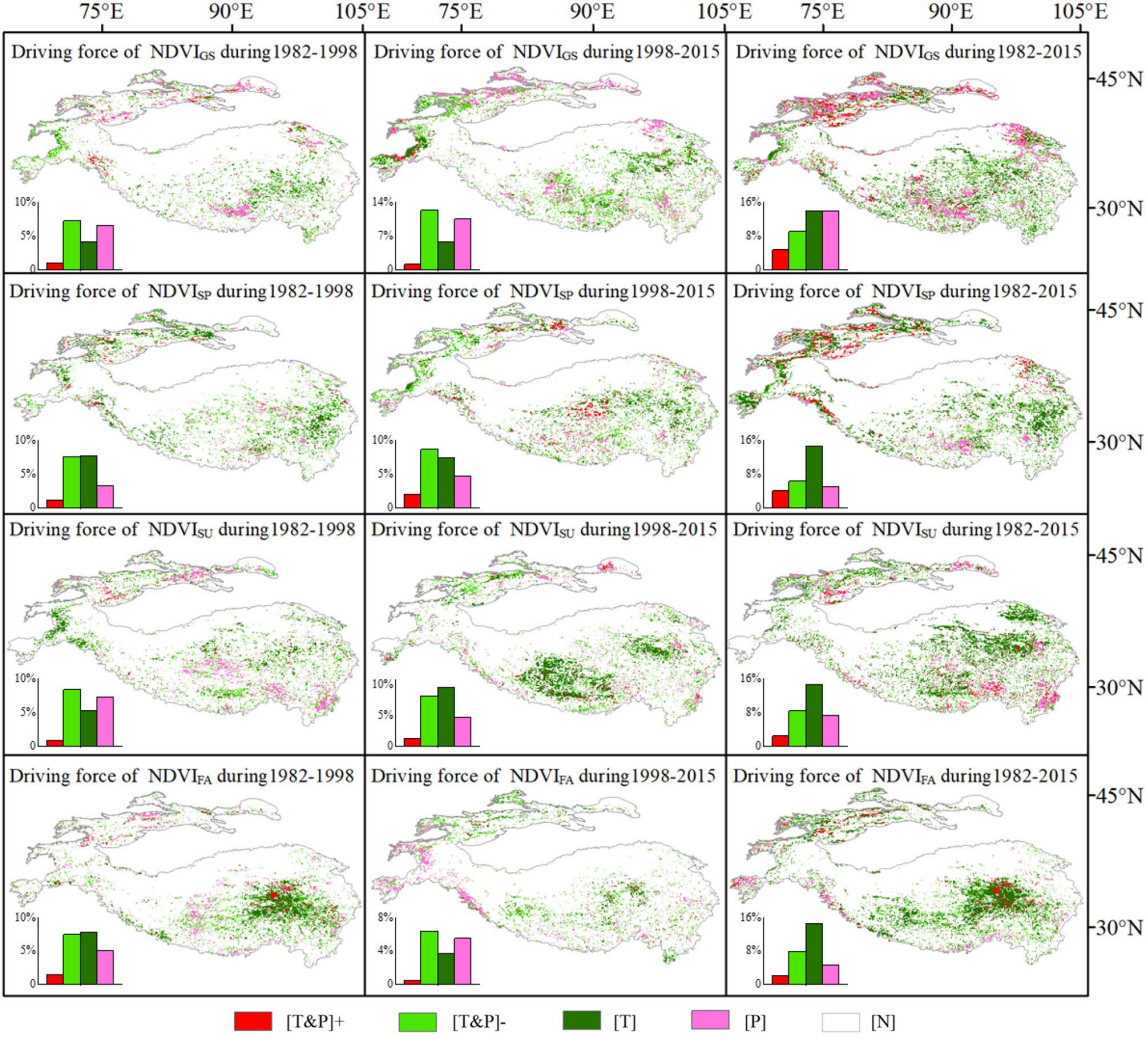
Spatial distribution of climate factors significantly affecting vegetation dynamics in HMA for growing seasons (GS), spring (SP), summer (SU) and fall (FA) from 1982 to 2015 (Generated by ArcGIS 10.3, URL: http://www.esri.com/software/arcgis/arcgis-for-desktop, and Origin 2020b, URL: https://www.originlab.com/).

Quantitative analysis results of driving force (Table 1) show that, among the three time spans, climate drive accounted for the largest proportion during the 1982–2015 growing seasons, with a value of 41.9% (Fig. 6). Temperature and precipitation drive accounted for 13.96% and 14.07% of the total vegetation change pixel, respectively. Prior to 1998, the proportion of vegetation dynamics driven by climate was 19.04%, while after 1998, vegetation browning was significant, the area driven by climate increased to 29.98%, and the proportion driven by precipitation also increased significantly. Temperature-driven changes were mainly located in northeastern HMA and the Hindu Kush area.

**Table 1:** Criteria for classifying dominant climate factors.

Driving factors	Criteria		
	r_NDVIT,P_	r_NDVIP,T_	R_NDVI,TP_
Driven by temperature [T]	t ≥ t_0.05_	t ≤ t_0.05_	F ≥ F_0.05_
Driven by precipitation [P]	t ≤ t_0.05_	t ≥ t_0.05_	F ≥ F_0.05_
Strongly driven by temperature and precipitation [T&P] +	t ≤ t_0.05_	t ≤ t_0.05_	F ≥ F_0.05_
Weakly driven by temperature and precipitation [T&P] −	t ≥ t_0.05_	t ≥ t_0.05_	F ≥ F_0.05_
Non-climate driving [N]			F ≤ F_0.05_

In terms of seasons, between 1982 and 1998, spring and fall were significantly temperature-driven, with proportions of 7.71% and 7.82%, respectively, distributed across the Tianshans and the central TP. The summer and overall growing seasons were markedly affected by precipitation, accounting for 7.25% and 6.54%, respectively. These areas were distributed in southern HMA and the eastern Tianshans. After 1998, the spring and summer were significantly temperature-driven, accounting for 7.46% and 9.61%, respectively, and were distributed in central and southern HMA. The fall and growing seasons, however, were significantly precipitation-driven, accounting for 5.53% and 10.53%, respectively, and distributed in southwestern HMA and the Tianshans. Therefore, we can assume that climate change in HMA is still the main influencing factor of regional vegetation dynamics.

## Discussion

### Reassessment of vegetation dynamics

Reassessment of the NDVI changes in vegetation in HMA under the condition of abrupt warming shows that the trend reversed from greening to browning after 1998. Previous studies have shown that with increases in temperature and precipitation over the past 30 years, the overall vegetation in HMA has a greening tendency^5,49^, whereas our results found that the vegetation has browned in the context of overall greening. Against a backdrop of increasing temperature and precipitation, the NDVI in HMA first increased at a rate of 0.012 per decade. Then, because of the continuous temperature increase and precipitation decrease, the NDVI trend reversed, decreasing at a rate of 0.005 per decade.

Therefore, the increase of temperature and precipitation makes the HMA vegetation greening, which is similar to the previous research results^50^. However, after the temperature jump, we found that the continuous warming and the decrease of precipitation are the key factors limiting the growth of vegetation, rather than the simple precipitation inhibiting the growth of vegetation. It shows that the dynamic change of vegetation under the background of global climate change is not simply dominated by temperature or precipitation, but influenced by them at different time scales.

Vegetation dynamics also show significant temporal and spatial differences. Temporally, spring fluctuations are the largest (but with the smallest proportion), while summer fluctuations are the smallest (but with the largest proportion). Further, our data show that the fall proportion is slightly higher than the one in spring, and that the fluctuation is relatively mild. The increase of temperature in spring and autumn will promote the extension of growing season and biomass accumulation by affecting the phenological process of vegetation, while the increase of temperature in summer will not promote the growth of vegetation, and even limit the growth of vegetation through plant physiological process and atmospheric environment.

Spatially, bearing in mind that the reversal of the vegetation trend is mainly located in the northwest and southeast of HMA, which may be explained by the fluctuation of the regional water deficit. Fluctuations in water deficit are caused by factors such as a jump in temperature and a reversal in precipitation. The reason is that there is abundant precipitation in these areas at the beginning, it can make up for the negative effect of warming in spring and summer, then promote vegetation growth together with temperature. Relevant studies suggest that the North Atlantic Oscillation (NAO) leads to the weakening of the mid latitude westerly wind and the influence of non-intrusive convective storms (CS_S_) events^51–53^, thus reducing the precipitation of HMA. At the same time, the latest research showed that the optimal temperature of HMA vegetation is far lower than that of ecosystems in other parts of the world. That is to say, when the precipitation base changes from humid to relatively dry, it intensifies the drought stress caused by warming and becomes the main factor limiting vegetation growth.

### Impacts of vegetation dynamic driving mechanism

**Figure 7:**
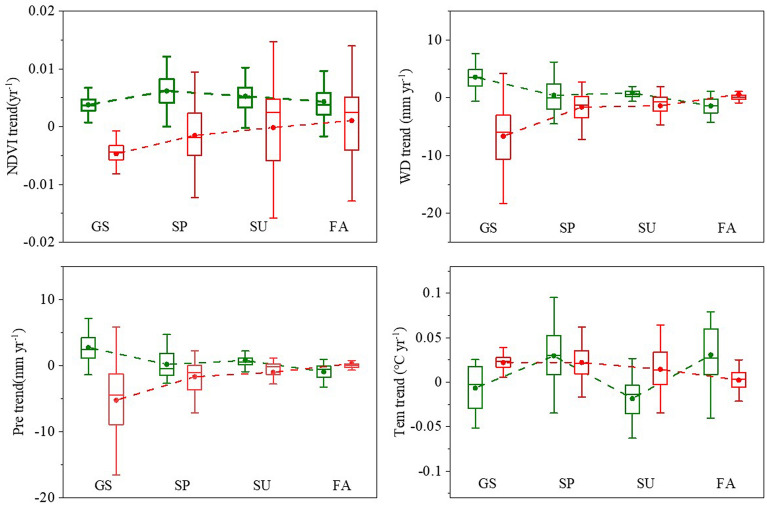
NDVI, temperature, precipitation and water deficit trend box chart of HMA for growth season, spring, summer and fall from 1982 to 2015. Green represents 1982 to 1998 and red represents 1998 to 2015. The box plot is drawn by the trend values of grid points passing the significance test (P < 0.001) (Generated by Origin 2020b, URL: https://www.originlab.com/).

The increase in temperature at high altitudes can help plants to photosynthesize and promote biomass accumulation^54^. At the same time, the increase in precipitation can improve water conditions in HMA and promote the greening of vegetation and the extension of the growing season^55^. This is because according to the data of the annual aridity index (the ratio of precipitation to potential evapotranspiration) and vegetation cover type, we can find that most areas of HMA are in a cold arid or semi-arid climate^50,56^. And there are mainly alpine meadows, grasslands, etc., which are sensitive to temperature and precipitation^57^. The rise of temperature provides favorable conditions for the growth of plants in cold and arid areas, but when the temperature exceeds the optimal temperature, it will bring adverse factors to the growth of vegetation^35,58^.However, we found that the continuous warming after the temperature jump in 1998 had negative side effects on plant growth. On the one hand, too high temperature will increase water evaporation^30^, reduce soil water content^59^, reduce primary productivity of HMA vegetation in arid climate^58^ and limit plant growth^35,57^. On the other hand, vegetation in cold and arid areas will have certain physiological response to high temperature, such as, the date of vegetation turning green in spring and falling leaves in autumn is advanced or delayed due to poor accumulated temperature^60^. HMA also experienced reverse changes in precipitation. Although the overall trend of humidification was maintained, it has changed from an upward to a downward trend (Fig. 7). It is generally believed that the increase of precipitation can alleviate the decrease of soil moisture and play a certain cooling process. That is to say, if the precipitation doesn’t increase or decrease, the continuous rise of temperature will have a great negative effect on the growth of vegetation. Therefore, when considering the attribution of vegetation dynamics in HMA, we can’t simply use the linear relationship to analyze the impact of temperature or precipitation on vegetation NDVI. We think that it is the combination of temperature and precipitation, that is to say, water deficit (which can represent the joint effect of energy and water) affects the vegetation dynamics. The coupling result of water deficit and NDVI confirmed our conjecture (Fig. 5).

The change trend of NDVI from 1982 to 1998 was positively correlated with temperature and precipitation, and the water deficit rate was positive, indicating that optimal temperature which is the suitable temperature for vegetation growth in HMA and precipitation increases effectively slowed down the water deficit and promoted vegetation greening. Precipitation was thus shown to be the main factor affecting vegetation greening. However, the tendency of NDVI from 1998 to 2015 was negatively correlated with temperature and positively correlated with precipitation. The water deficit rate was negative, indicating that warming had begun to restrict plant growth, and the reduction of precipitation further aggravated the water deficit, which eventually resulted in the transformation of vegetation into a browning tendency.

Although these results are in line with those of existing studies, earlier investigations mostly considered temperature and precipitation as the main factors^61,62^. Through our analysis of trend values (Fig. 7), we believe that the greening and browning of HMA vegetation is not simply linear with changes in temperature and precipitation, but is instead manifested as a dual effect of temperature and a superimposed effect of precipitation. Previous studies have confirmed the dual effect of temperature and precipitation^31,35,63–65^. Shen et al.^66^ found that the length and range of plant growth in high altitude areas are mainly limited by low temperature^26^. At the same time, the availability of water will greatly limit the growth of HMA vegetation^67,68^. Unfortunately, most studies have not explained the dual mechanism of temperature and precipitation. Specifically, our results show that warming firstly promotes the greening of HMA vegetation with lower average temperature, but the continuous warming after the temperature jump increased the degree of water deficit and restricted vegetation greening. Meanwhile, precipitation was first increased to provide suitable water conditions for vegetation greening, but precipitation fell after the reversal. This further aggravated the water stress caused by the continuous temperature rise, eventually leading to the reversal of HMA vegetation from greening to browning.

### Impacts on future vegetation-climate feedback

Climate change is a major threat to biodiversity and ecosystems^69^. Ecosystem changes characterized by vegetation dynamics are not only affected by climate change but also sensitive to human activities^70,71^. Although excessive human intervention has led to a substantial reduction in vegetation coverage^72^, large-scale artificial ecological projects can offset the browning trend of vegetation^32^. For example, in the past few decades, overgrazing and climate change in HMA have led to 90% of alpine grassland degradation^73–75^. The national ecological project of "Returning Grazing Land to Grassland" implemented by the Chinese government since 2003 has played a great role in vegetation restoration and carbon sequestration in HMA^76,77^. In addition, the ecological protection and construction project of three river source implemented in 2005 has played a positive role in water conservation, grassland coverage and productivity improvement in HMA^78,79^. Compared with other regions, HMA is increasingly drawing the attention of researchers and scholars, as it generally sees less human activity and the development of vegetation dynamics is thus mostly affected only by natural conditions^80–82^. As mentioned earlier, climate change is the main driving force of vegetation dynamics in HMA, and this mechanism is dual and superimposed, accounting for about 20–40% of the driving factors of vegetation dynamics. These results are slightly larger than those of Zhu's analysis of global vegetation drivers^6^.

Additionally, the greening period of regional vegetation has been shown to be typically accompanied by a cooling and humidity trend, while the browning period is accompanied by a warming and drying trend^83,84^. This shows that while vegetation responds to climate change, it also plays a role in regulating climate change in some way. It is indispensable that we should pay more attention to the changes of vegetation phenological indicators (such as turning green and withering) when we attribute seasonal vegetation dynamics, because it determines the growth cycle and biomass accumulation of vegetation^67^. Due to the complexity of the climatology, it is necessary to consider the hysteretic effects of temperature and precipitation and the asymmetric effects of daytime and nighttime temperature^85,86^ when making detailed attribution of vegetation dynamic information (such as phenology and biomass)^63,66^, because it will help us better understand the response and feedback mechanism of vegetation change to climate change. Although the complete attribution of vegetation dynamics and the precise quantification of the feedback mechanism of vegetation dynamics to climate change have become important research directions for assessing and predicting ecosystem response and feedback to climate change^87^, relevant definitive research is still lacking^55^. Therefore, the research on the mutual feedback mechanism of HMA’s vegetation dynamics and climate change are open for future researchers.

## Materials and methods

### NDVI data

The GIMMS NDVI3g data set comes from the National Aeronautics and Space Administration (NASA, https://ecocast.arc.nasa.gov/data/pub/gimms/). It is currently the longest NDVI global data product (1982–2015), with a spatial resolution of 8 km × 8 km and a temporal resolution of 15 d. This data set has been widely used in the study of vegetation dynamics at various temporal and spatial scales^88,89^. Pixels with NDVI values greater than 0.1 are used for analysis^90^, and the growing season (GS) is defined as May–October, according to relevant research literature^49^. The Maximum Value Composite (MVC) method is utilized to construct the monthly and growing season datasets. To determine the climate characteristics of the study area, the NDVI monthly data are collected into seasonal data sets by using the Average Value Composite (AVC) method and the standards of spring (May–June, SP), summer (July–August, SU), and fall (September–October, FA).

### Meteorological data

The CRU_TS4.04 version (released in April 2020, https://doi.org/10.1038/s41597-020-0453-3) meteorological data set was developed by the Climatic Research Unit (CRU) of the University of East Anglia and the Hadley Centre (British Meteorological Office). The latest version of the data better improves the traceability between each grid value and the input observation value. From CRU_TS4.04, our study selects monthly temperature, precipitation and potential evapotranspiration data sets from 1982 to 2015, with a spatial resolution of 0.5° × 0.5°.

### Data aggregation

Water deficit can be calculated pixel-by-pixel using precipitation and potential evapotranspiration data. The formula is:

1 $$WD_{i} = PRE_{i} - PET_{i}$$

In the formula, *WD*_*i*_ is the water deficit value based on pixel synthesis, such that the larger the value, the better the water condition. Otherwise, it means the water stress is serious.

### Trend analysis

We established a linear regression equation of annual average temperature (*y*, °C), precipitation (*y*, mm), NDVI (*y*), and time series (*x*, annual), as follows:

2 $$y_{i} = \alpha + \beta (x_{i} - \overline{x}) + e_{i}$$

where $\overline{x} = \frac{1}{N}\mathop \sum \limits_{i = 1}^{N} x_{i}$, ${ }\left( {x_{i} - \overline{x}} \right)$ is the anomaly; *e*_*i*_ is the random error; α and β are the linear fitting intercept and slope, respectively, by the least squares method; and *y*_*i*_ is the time series value of each pixel.

### Correlation analysis

It can describe the degree of correlation between variables, and pixel-by-pixel calculation can analyze variables from multiple dimensions in time and space. The formula is:

3 $$R_{xy} = \frac{{\sum\nolimits_{i = 1}^{n} {(x_{i} - \overline{x})(y_{i} - \overline{y})} }}{{\sqrt {\sum\nolimits_{i = 1}^{n} {(x_{i} - \overline{x})^{2} \sum\nolimits_{i = 0}^{n} {(y_{i} - \overline{y})^{2} } } } }}$$

where *R*_*xy*_ is the simple correlation coefficient of variables *x* and *y*; *x*_*i*_ is the value of the independent variable in the *i*th year; $\overline{x}$ is the mean value of the independent variable; *y*_*i*_ is the value of the dependent variable in the *i*th year; $\overline{y}$ is the mean value of the dependent variable; and *n* = 1,2…,34.

### Partial correlation analysis

It can eliminate the interference of other variables and describe the degree of correlation between two variables more accurately. The formula is as follows:

4 $$R_{xy,z} = \frac{{R_{xy} - R_{xz} R_{yz} }}{{\sqrt {(1 - R_{xz}^{2} )(1 - R_{xy}^{2} )} }}$$

where *R*_*xy,z*_ is the difference between the dependent variable and the independent variable after the partial correlation coefficient control variable. If we use the *t* test to determine the significance of the partial correlation coefficient, the calculation formula is as follows:

5 $$t = \frac{{R_{{xy,z^{{\sqrt {n - m - 1} }} }} }}{{\sqrt {1 - R_{xz,y}^{2} } }}$$

where *t* is the statistic, *R*_*xy,z*_ is the partial correlation coefficient, *n* is the number of samples, and *m* is the number of independent variables.

### The multiple correlation coefficient analysis

It can describe the degree of correlation between a variable and a set of variables. The formula is as follows:

6 $$R_{x,yz} = \sqrt {1 - (1 - R_{xy}^{2} )(1 - R_{xz,y}^{2} )}$$

We use the *F* test to measure the significance of the multiple correlation coefficient, with the calculation formula as follows:

7 $$F = \frac{{R_{x,yz}^{2} }}{{1 - R_{x,yz}^{2} }} \times \frac{n - k - 1}{k}$$

where *n* is the number of samples and *k* is the number of independent variables.

### Autocorrelation function analysis

Autocorrelation function (ACF) analysis is a method to judge the properties of time and space series. Through autocorrelation analysis, we can reveal the attributes and properties of time series. This is the basis of time series regression analysis and correlation analysis. The formula is as follows:

8 $$r_{k} = \frac{{\sum\limits_{t = 1}^{n - k} {(x_{t} - \overline{x}} )(x_{t + k} - \overline{x})}}{{\sum\limits_{t = 1}^{n} {(x_{t} - \overline{x}} )^{2} }}$$

where *t* is the time series, *k* is the time delay, *n* is the length of the sample path, *x*_*t*_ is the *t*th value, and $\overline{x}$ is the mean value of the variable. For the random sequence, except for the 0-lag point without information, the other ACF values should change within the positive and negative double standard error band. In this case, the variable sequence has no autocorrelation, or the autocorrelation is not significant. It may be correlated with other variables and it can be studied by correlation analysis (formula 3). On the contrary, the original sequence of variables should be preprocessed. Then the correlation analysis was carried out^91^.

## Supplementary Information

Supplementary Information.
