# Supplementary material for: Continuous warming shift greening towards browning in the Southeast and Northwest High Mountain Asia
Source: Sci Rep. 2021 Sep 9;11:17920. doi: 10.1038/s41598-021-97240-4 (PMC8429466; doi:10.1038/s41598-021-97240-4)
Supplement: Supplementary file 1 — Supplementary Information. [file 41598_2021_97240_MOESM1_ESM.docx]

**Supplementary Materials**


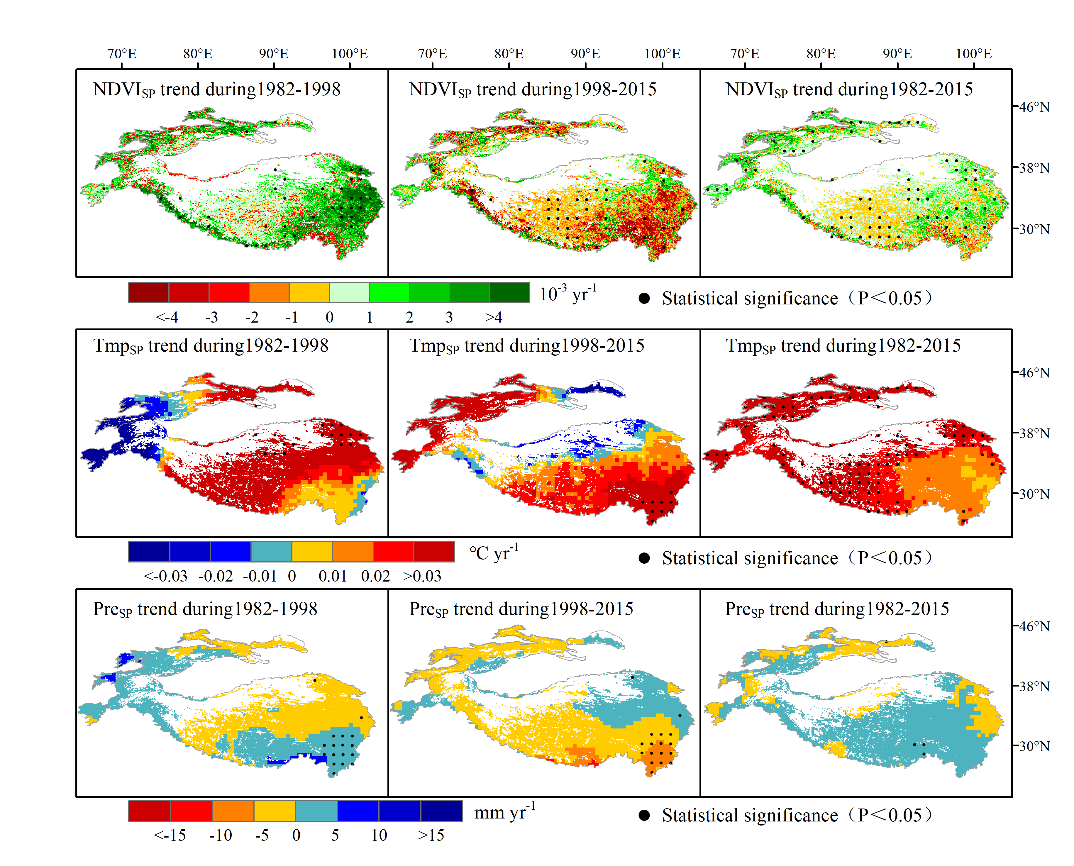


**Figure S1.** Spatial distribution of spring (May-June) NDVI trends for air temperature and precipitation in HMA from 1982 to 2015. Note that regions with black dots indicate that trend values are statistically significant (p < 0.05) (Generated by ArcGIS 10.3, URL: http://www.esri.com/software/arcgis/arcgis-for-desktop).


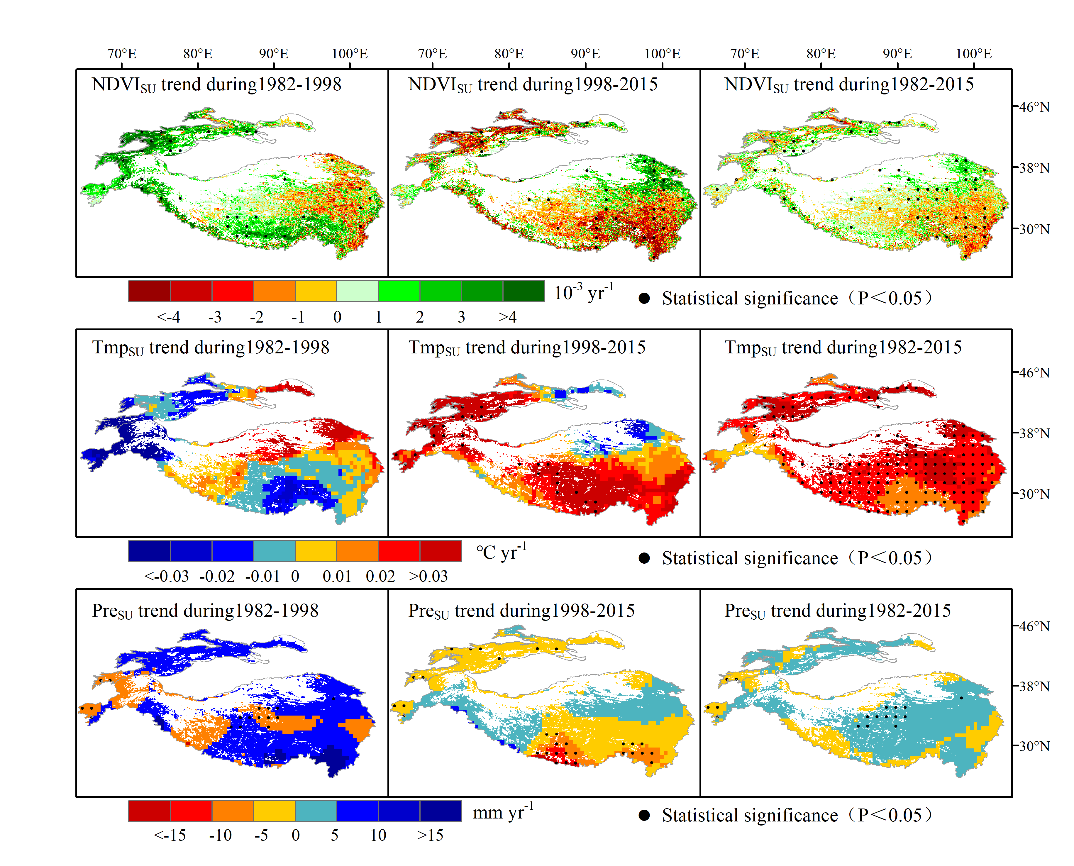


**Figure S2.** Spatial distribution of summer (July-August) NDVI trends for air temperature and precipitation in HMA from 1982 to 2015. Note that regions with black dots indicate that trend values are statistically significant (p < 0.05) (Generated by ArcGIS 10.3, URL: http://www.esri.com/software/arcgis/arcgis-for-desktop).


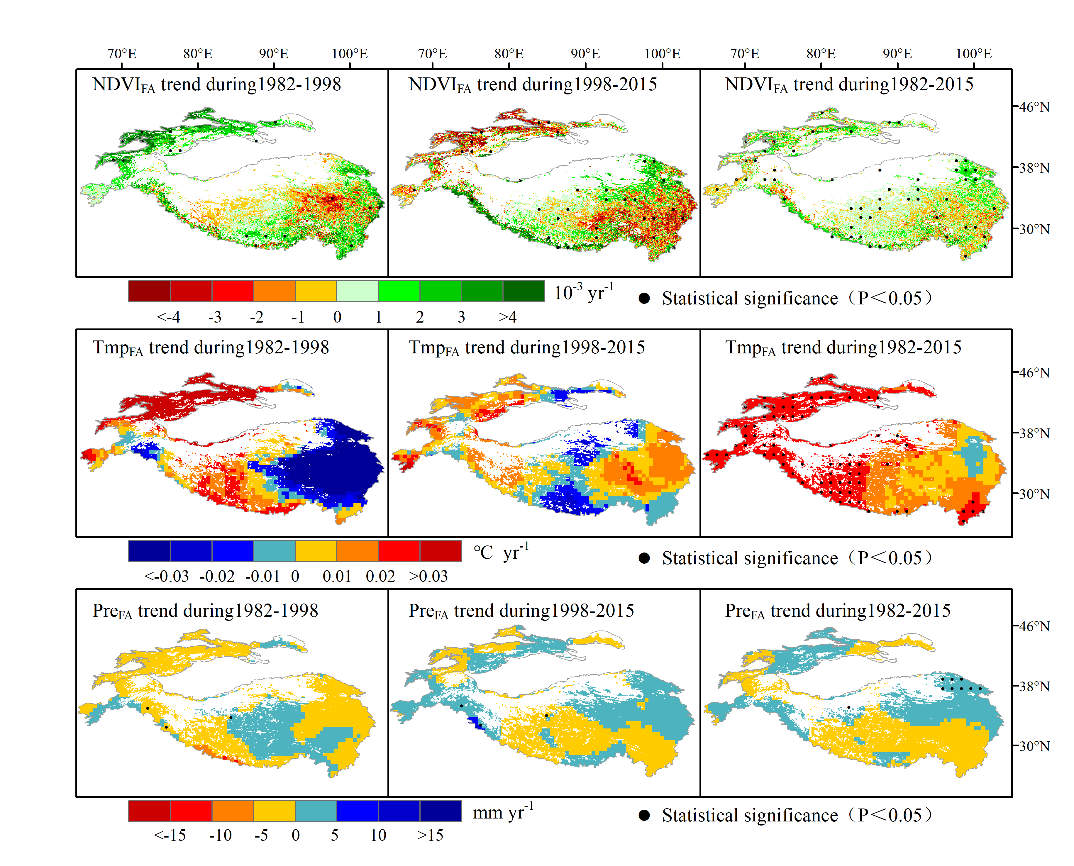


**Figure S3.** Spatial distribution of fall (September-October) NDVI trends for air temperature and precipitation in HMA from 1982 to 2015. Note that regions with black dots indicate that trend values are statistically significant (p < 0.05) (Generated by ArcGIS 10.3, URL: http://www.esri.com/software/arcgis/arcgis-for-desktop).
